# What internal medicine attendings talk about at morning report: a multicenter study

**DOI:** 10.1186/s12909-023-04057-y

**Published:** 2023-02-02

**Authors:** Jeffrey W. Redinger, Daniel B. Heppe, Tyler J. Albert, Paul B. Cornia, Kirsha S. Gordon, Cherinne Arundel, Joel M. Bradley, Laura M. Caputo, Jonathan W. Chun, Jessica E. Cyr, Erik T. Ehlers, Michelle M. Guidry, Anand D. Jagannath, Brian K. Kwan, James D. Laudate, Christine A. Mitchell, Andrea C. Smeraglio, Joseph R. Sweigart, Matthew G. Tuck, Craig G. Gunderson

**Affiliations:** 1 VA Puget Sound Health Care System, 1660 S Columbian Way, Seattle, WA 98108 USA; 2 Department of Medicine, University of Washington School of Medicine, Seattle, WA, USA; 3 VA Eastern Colorado Health Care System, Aurora, CO, USA; 4 Department of Medicine, University of Colorado School of Medicine, Aurora, CO, USA; 5 VA Connecticut Health Care System, West Haven, CT, USA; 6 Department of Medicine, Yale University School of Medicine, New Haven, CT, USA; 7 Washington, DC VA Medical Center, Washington, DC, USA; 8 Department of Medicine, George Washington University School of Medicine and Health Sciences, Washington, DC, USA; 9 Department of Medicine, Georgetown University School of Medicine, Washington, DC, USA; 10 White River Junction VA Medical Center, White River Junction, VT, USA; 11 Department of Medicine, Geisel School of Medicine at Dartmouth, Hanover, NH, USA; 12 Durham VA Health Care System, Durham, NC, USA; 13 Department of Medicine, Duke University School of Medicine, Durham, NC, USA; 14 VA Palo Alto Health Care System, Palo Alto, CA, USA; 15 Stanford University School of Medicine, Stanford, CA, USA; 16 VA Pittsburgh Healthcare System, Pittsburgh, PA, USA; 17 Department of Medicine, University of Pittsburgh School of Medicine, Pittsburgh, PA, USA; 18 Omaha VA Medical Center, Omaha, NE, USA; 19 Department of Internal Medicine, University of Nebraska College of Medicine, Omaha, NE, USA; 20 Southeast Louisiana Veterans Health Care System, New Orleans, LA, USA; 21 Tulane University School of Medicine, New Orleans, LA, USA; 22 VA Portland Health Care System, Portland, OR, USA; 23 Department of Medicine, Oregon Health & Science University, Portland, OR, USA; 24 San Diego VA Medical Center, San Diego, CA, USA; 25 Department of Medicine, University of California San Diego School of Medicine, San Diego, CA, USA; 26 Lexington VA Health Care System, Lexington, KY, USA; 27 Department of Internal Medicine, University of Kentucky College of Medicine, Lexington, KY, USA

**Keywords:** Medical education, Graduate medical education, Morning report, Internal medicine residency

## Abstract

**Background:**

Morning report is a core educational activity in internal medicine resident education. Attending physicians regularly participate in morning report and influence the learning environment, though no previous study has described the contribution of attending physicians to this conference. This study aims to describe attending comments at internal medicine morning reports.

**Methods:**

We conducted a prospective, observational study of morning reports conducted at 13 internal medicine residency programs between September 1, 2020, and March 30, 2021. Each attending comment was described including its duration, whether the comment was teaching or non-teaching, teaching topic, and field of practice of the commenter. We also recorded morning report-related variables including number of learners, report format, program director participation, and whether report was scripted (facilitator has advance knowledge of the case). A regression model was developed to describe variables associated with the number of attending comments per report.

**Results:**

There were 2,344 attending comments during 250 conferences. The median number of attendings present was 3 (IQR, 2–5). The number of comments per report ranged across different sites from 3.9 to 16.8 with a mean of 9.4 comments/report (SD, 7.4). 66% of comments were shorter than one minute in duration and 73% were categorized as teaching by observers. The most common subjects of teaching comments were differential diagnosis, management, and testing. Report duration, number of general internists, unscripted reports, and in-person format were associated with significantly increased number of attending comments.

**Conclusions:**

Attending comments in morning report were generally brief, focused on clinical teaching, and covered a wide range of topics. There were substantial differences between programs in terms of the number of comments and their duration which likely affects the local learning environment. Morning report stakeholders that are interested in increasing attending involvement in morning report should consider employing in-person and unscripted reports. Additional studies are needed to explore best practice models of attending participation in morning report.

## Background

Morning report is a foundational educational conference in graduate medical education. Nearly all internal medicine residencies hold morning report and residents consistently rate the conference highly [1–4]. Over time, the purpose and structure of morning report have evolved [5, 6]. Morning report traditionally consisted of residents presenting overnight admissions to faculty, largely for quality control and resident evaluation [7–12]. Contemporary studies have found that morning report has since become a venue for delivering case-based education, with most conferences facilitated by a chief resident using electronic presentation slides to present patient information and teaching points [4–6, 12].

These changes have been accompanied by heterogeneity in the role of the attending physician in morning report. Two recent surveys have helped delineate resident and program director expectations. A 2021 survey of residents at ten internal medicine programs concluded that the duration and content of attending commentary influences residents’ perceptions of morning report quality but found that only 61% of residents desired faculty attendance [13]. Similarly, a 2022 survey of internal medicine program directors found contrasting opinions on how often attending physicians should make comments during morning report, with some praising frequent teaching by faculty and others striving to maximize resident-driven education with less faculty commentary [14]. A 2013 literature review made note of heterogeneity in the purpose, structure, and scope of morning report practices, and suggested a common aim of morning report as a forum for resident professional identity formation with faculty acting as supportive educators [5].

Though residents and program directors perceive attending physicians as influential to the morning report learning environment, there is a surprising paucity of data describing faculty teaching practices during this conference. Future research on best practice models of faculty participation in morning report should be guided by an initial description of current practices. Therefore, we conducted an observational study describing the content and frequency of comments made by attending physicians during morning report.

## Methods

### Setting and participants

We observed internal medicine morning report at thirteen geographically varied internal medicine residency programs in the United States. Sites were recruited from the Veteran Affairs National Academic Hospitalist Work Group [15]. The thirteen sites were in Seattle Washington, Portland Oregon, Palo Alto California, San Diego California, Denver Colorado, New Orleans Louisiana, Omaha Nebraska, Pittsburgh Pennsylvania, Durham North Carolina, Lexington Kentucky, Washington, D.C., West Haven Connecticut, and White River Junction Vermont. Each hospital has at least one Accreditation Council for Graduate Medical Education accredited internal medicine residency program with between 64 and 194 residents (average 131). The Veterans Affairs Central Institutional Review Board (IRB) determined that the study was exempt from local Institutional Review Board review because it involved a normal educational practice in a common setting. Local IRB exemption procedures were followed at each site. Sites that had virtual morning reports which involved the university affiliate also received university IRB exemptions.

### Study design and data collection

We conducted a prospective observational study of the comments made by attending physicians during internal medicine morning report. Data was collected between September 1, 2020 and March 30, 2021. A standardized data collection tool was iteratively developed during monthly conference calls by the study investigators. Standardized instructions were disseminated to each site investigator. At each site, investigators observed a convenience sample of morning reports and recorded a range of variables including whether the report was in-person or virtual or a combination. We collected data about the format, structure, and content of each observed conference, as well as the number of participants. Additional variables included conference duration, number of learners and level of training, number of attendings presents and field of practice, number of chief residents present, case diagnosis if known, whether the format was scripted (facilitator has advance knowledge of the case) or unscripted (facilitator has no advance knowledge of the case) [16] and use of electronic presentation slides. Attending field of practice was recorded as hospitalist if the attending’s primary professional focus was medical care of the hospitalized patient, or general internist if the attending’s primary professional focus was medical outpatient care. If a conference had a virtual component, site investigators recorded the number of virtual groups that attended, defined by the number of unique virtual logins. Attending comments were coded as in-person, virtual audio/video, or virtual chat. For each comment made by an attending, site investigators recorded the approximate duration, whether it was prompted by the facilitator, whether it prompted further discussion, a qualitative description, and classified the comment as teaching or non-teaching. A comment was classified as prompted by the facilitator if attending commentary was requested by the facilitator directly prior to the comment. The total number of comments per report was also recorded. Each investigator further coded comments by type of content. Site investigators were instructed to count themselves in data collection only if they normally would have participated in the conference. Comments made during morning report by these investigators were included in the analysis.

### Statistical analysis

Report and comment level variables were described using frequencies for categorical variables and medians and interquartile ranges for continuous variables. Differences between sites for the number of attending comments per report were analyzed using one-way ANOVA and Pearson’s chi-squared for frequency of comments greater than one minute.

Attending comment descriptions coded as teaching comments were further analyzed by teaching category for content by two authors (jr,cg). Comments coded as differential diagnosis were divided into syndrome and specific diagnoses: for example, rash and endocarditis. Comments coded as management were similarly divided into general management category and specific disease: for example, medications and hepatic encephalopathy. Lastly, comments coded as diagnostic testing were divided into testing category and specific tests: for example, imaging and computed tomography of the chest.

A negative binomial regression model was employed to examine the association between report level variables and the number of comments per report. The negative binomial regression model was employed because the outcome of interest (number of comments, which was a discrete count) was not normally distributed, and the conditional variance exceeded the conditional mean. This difference implies that over-dispersion was present, rendering a Poisson distribution inappropriate and the negative binomial regression the best option [17]. The final model was determined using a threshold of *p* < 0.10 for variables from univariate analysis. Statistical analyses were conducted via SAS, version 9.4 (SAS Institute Inc, Cary, NC) and Stata/IC, version 16.1 (StataCorp, College Station, TX).

## Results

### Site characteristics

We observed 250 morning reports from 13 residency programs. An overview of the sites is shown in Table 1. Most sites observed at least 20 reports. Three sites held the majority of conferences in-person. The remaining sites largely held virtual reports. Unscripted reports were the predominant format at five sites. Four sites held only scripted reports, and the remaining sites had a mix of unscripted and scripted reports.

**Table 1:** Overview of participating sites

Location	Reports Observed No. (%)	Comments No. (%)	In-person report No. (%)	Unscripted reports No. (%)	Comments per Report, Mean (SD)^a^	Comments > 1 min No.(%)^b^
**Midwest**						
**Site #1**	13	121 (5)	1/13 (8)	13/13 (100)	9.3 (3.1)	82/121 (68)
**Site #2**	17	285 (12)	2/17 (12)	13/17 (76)	16.8 (7.1)	74/277 (27)
**Northeast**						
**Site #3**	20	231 (10)	19/20 (95)	0	11.6 (6.2)	28/231 (12)
**Site #4**	17	73 (3)	3/17 (18)	4/17 (24)	4.3 (3.5)	6/21 (29)
**Site #5**	25	188 (8)	0/25 (0)	0	7.5 (5.0)	40/156 (26)
**Site #6**	25	132 (6)	0/25 (0)	5/25 (20)	5.3 (4.2)	29/100 (29)
**South**						
**Site #7**	18	289 (12)	15/18 (83)	16/18 (89)	16.1 (7.0)	130/287 (45)
**Site #8**	16	77 (3)	16/16 (100)	9/16 (56)	4.8 (2.4)	34/77 (44)
**West**						
**Site #9**	16	62 (3)	1/16 (6)	3/16 (19)	3.9 (2.7)	5/32 (16)
**Site #10**	20	193 (8)	0/20 (0)	18/20 (90)	9.6 (6.0)	22/41 (54)
**Site #11**	20	303 (13)	0/20 (0)	0	15.1 (11.8)	10/77 (13)
**Site #12**	23	290 (12)	8/23 (35)	0	12.6 (6.1)	83/239 (35)
**Site #13**	20	100 (4)	0/20 (0)	6/20 (30)	5 (5.8)	28/76 (37)
**Totals**	250	2,344	65/250 (26)	87/250 (35)	9.4 (7.4)	571/1,735 (25)

^a^Between group comparison, F = 11.05, *p* < .0001, ^b^ Χ^2^ (12, *N* = 1,735) = 172.6, *p* < .001

### Description of morning reports

In-person reports accounted for 26% of observed conferences. The remaining reports were approximately equally divided between virtual at a single site (21%), virtual across multiple sites (22%), or a hybrid conference with virtual and in-person attendees (30%). Most conferences were attended by residents (97%), interns (87%) and medical students (72%). Conferences were predominantly case-based (83%) with a small number of journal club (9%), lecture-based (2%), and game-based (2%) reports. Conferences were led primarily by chief residents (81%) with the remainder led by attending physicians (10%) or second- or third-year residents (9%). During case-based reports, clinical details were frequently provided by a resident (70%) and less often by a chief resident (20%) or intern (6%). Most conferences were scripted (65%) and the median morning report duration was 50 min (IQR, 42 – 60).

The median number of attendings present at morning report was 3 (IQR, 2.0 – 5.0), most of whom were hospitalists (median 2, IQR, 1.0 – 3.0). A program director or associate/assistant program director was present at 59% of conferences. The median number of learners was 15 (IQR, 7.0 – 26.0). During conferences that included virtual learners, there was a median of 3 virtual groups that participated (IQR, 0 – 6.0).

### Description of attending comments

The mean number of comments per report was 9.4, with a range of 3.9 to 16.8 comments per report at different sites. After excluding chat, 33% of comments were longer than one minute, with a range between 12 and 68% across sites. Both the number of comments per report (*p* < 0.001) and proportion greater than one minute (*p* < 0.001) were statistically significantly different between sites. Approximately equal proportions of comments were made by hospitalists, general internists, and internal medicine subspecialists (Table 2). Similar numbers of in-person (38%; 889/2,333) and virtual audio/video (36%, 847/2,333) comments were observed, while virtual chat box comments (26%, 597/2,333) were observed less often. Most comments were brief and, excluding virtual chat, 66% were shorter than one minute. Forty one percent of comments prompted further discussion amongst trainees. Twenty one percent of comments were prompted by the facilitator. During conferences facilitated by attending physicians, the rate of prompted attending comments was fifteen percent. Seventy three percent of all comments were categorized as educational by observers with the top teaching topics being differential diagnosis (21%), management (14%), and testing (10%). The most common categories of non-teaching comments were questions (50%), jokes (14%), and past clinical experiences (9%).

**Table 2:** Description of attending comments

Characteristic	Values^a^
**Type of Attending Commenter, No (%)**	
Hospitalist	846/2,333 (36)
General Internist	699/2,333 (30)
Internal Medicine Subspecialist	769/2,333 (33)
Non-internist	19/2,333 (0.8)
**Commenter, PD or APD, No (%)**	
Yes	414/2,333(18)
No	1,919/2,333 (82)
**Comments per Report, Mean (SD)**	9.4 (7.4)
**Comment Duration, No (%)**^**b**^	
< 10 s	376/1,735 (22)
10–60 s	788/1,735 (45)
1–2 min	318/1,735 (18)
2–3 min	147/1,735 (6)
3–4 min	50/1,735 (3)
4–5 min	14/1,735 (1)
> 5 min	44/1,735 (3)
**Comment In-person or Virtual Audio/Video or Chat, No (%)**	
In-person	889/2,333 (38)
Virtual, audio	847/2,333 (36)
Virtual, chat	597/2,333 (26)
**Did Comment Prompt discussion, No (%)**	
Yes	945/2,321 (41)
No	1,376/2,321 (59)
**Comment Prompted by Facilitator, No (%)**	
Yes	476/2,321 (21)
No	1,845/2,321 (79)
**Did Comment Teach, No (%)**	
Yes	1,705/2,333 (73)
No	628/2,333 (27)
**Type of Teaching, No (%)**	
Differential Diagnosis	361/1,727 (21)
Management	245/1,727 (14)
Testing	165/1,727 (10)
Labs	137/1,727 (8)
Clinical Reasoning	122/1,727 (7)
Physician Exam	116/1,727 (7)
Pathophysiology	112/1,727 (6)
History	99/1,727 (6)
Evidence Appraisal	92/1,727 (5)
Imaging	65/1,727 (4)
High Value Care	22/1,727 (1)
Patient Safety	19/1,727 (1)
EKG	13/1,727 (0.7)
Ethics	10/1,727 (0.6)
Social Determinants of Health	8/1,727 (0.5)
Medical Education	5/1,727 (0.3)
Multiple	87/1,727 (5)
**Type of Non-teaching Comment, No (%)**	
Question	304/611 (50)
Joke	83/611 (14)
Past Experience	52/611 (9)
Next Step in Care	23/611 (4)
Announcement	22/611 (4)
Criticism of patient care	9/611 (1)
Other	118/611 (19)

*PD* Residency Program Director, *APD* Associate Program Director, *EKG* Electrocardiogram

^a^Number of comments less than 2344 because of missing data or subgroup analysis

^b^Comment durations not including virtual chat comments

### Variables associated with number of comments

In univariate analysis, in-person reports, report duration, number of attendings, number of general internists, and attending report-leaders were all associated with increased number of comments (Table 3). Increased number of learners, the presence of a program director or associate/assistant program director and scripted reports were all associated with fewer attending comments. In multivariate analysis, in-person reports, report duration, and number of general internists were all associated with increased comments, while scripted reports remained associated with significantly fewer comments. In-person reports were associated with 57% more attending comments, while scripted reports were associated with 28% fewer attending comments. Each additional non-hospitalist general internist present was associated with 26% increased comments. Number of attendings was not included in the adjusted model because it was collinear with number of general medicine attendings.

**Table 3:** Unadjusted and adjusted determinants of attending comments per report

	**Unadjusted IRR (95% CI)**	***p*-value**	**Adjusted IRR (95% CI)**	***p*-value**
In-person report	1.58 (1.30, 1.92)	< 0.001	1.57 (1.23, 2.01)	0.0003
Number of learners	0.99 (0.99, 1.00)	0.06	1.00 (0.99, 1.00)	0.25
Case-based	1.24 (0.94, 1.63)	0.14		
Journal club	1.02 (0.72, 1.45)	0.90		
Lecture format	0.85 (0.41, 1.77)	0.67		
Chief resident presents	0.89 (0.68, 1.16)	0.40		
Resident presents	1.10 (0.87, 1.38)	0.44		
Scripted report	0.76 (0.62, 0.93)	0.01	0.72 (0.58, 0.91)	0.005
Report duration	1.02 (1.01, 1.03)	0.003	1.02 (1.00, 1.03)	0.01
Diagnosis known	0.91 (0.66, 1.26)	0.57		
Use of ppt	0.89 (0.70, 1.13)	0.33		
Number of ppt	1.00 (0.99, 1.01)	0.57		
Number attendings	1.04 (1.00, 1.08)	0.07		
Number of hospitalists	1.03 (0.97, 1.10)	0.32		
Number of gen med	1.33 (1.16, 1.52)	< 0.001	1.26 (1.09, 1.46)	0.002
Number of specialists	0.99 (0.92, 1.07)	0.78		
PD or APD	0.72 (0.59, 0.88)	0.001	1.03 (0.81, 1.31)	0.80
Chief resident leads	0.94 (0.73, 1.21)	0.61		
Attending leads	1.50 (1.08, 2.09)	0.02	0.94 (0.61, 1.46)	0.80

*Ppt* Electronic presentation slides, *Gen med* General medicine attendings, *PD* Residency Program Director, *APD* Associate/Assistant Program Director

The most common categories of teaching comments are shown in Table 4 and include differential diagnosis, management, and testing. The most common differential diagnosis syndromes discussed by attendings were delirium, polyarthritis, and rash. The most common specific diagnoses that attendings discussed were tuberculosis, endocarditis, histoplasmosis, adrenal insufficiency, and lymphoma. The most common categories of management were medications, diagnostic approach, and indications for surgery, while the most common specific diseases under management were hyponatremia, hepatic encephalopathy, and COVID-19. The top three categories of diagnostic testing comments included imaging, biopsies, and serum chemistry studies. The most common specific tests discussed were d-dimer, IFN-ү release assay, computed tomography of the chest, urinalysis, and arterial blood gas.

**Table 4:** Most common comments by teaching category

**Differential Diagnosis**			
**Syndrome**	**Number (%)**	**Disease**	**Number (%)**
Delirium	12/200 (6)	Tuberculosis	8/152 (5)
Polyarthritis	10/200 (5)	Endocarditis	7/152 (5)
Rash	9/200 (5)	Histoplasmosis	5/152 (3)
Shortness of Breath	8/200 (4)	Adrenal Insufficiency	4/152 (3)
Edema	6/200 (3)	Lymphoma	4/152 (3)
Hyponatremia	6/200 (3)	Abscess	3/152 (2)
Pulmonary Infiltrate	6/200 (3)	Aortic Dissection	3/152 (2)
Abnormal LFTs	5/200 (3)		
Abdominal Pain	5/200 (3)		
Neck mass	5/200 (3)		
**Management**			
**Management Category**		**Disease**	
Medications	101/190 (53)	Hyponatremia	10/229 (4)
Diagnostic approach	21/190 (11)	Hepatic encephalopathy	7/229 (3)
Indication for Surgery	18/190 (9)	COVID-19	7/229 (3)
Disposition	8/190 (4)	Delirium	7/229 (3)
Fluid management	5/190 (3)	Pneumothorax	6/229 (3)
Transfusion	4/190 (2)	VTE	6/229 (3)
Respiratory Failure	4/190 (2)	DKA	6/229 (3)
Prediction Scores	4/190 (2)	Variceal Hemorrhage	6/229 (3)
**Diagnostic Testing**			
**Testing Category**		**Specific Test**	
Imaging	22/128 (17)	D-dimer	5/124 (4)
Biopsy	13/128 (10)	IFN-γ release assay	5/124 (4)
Serum Chemistry	13/128 (10)	CT Chest	4/124 (3)
Peripheral Smear	10/128 (8)	Urinalysis	4/124 (3)
Lumbar Puncture	8/128 (6)	Arterial blood gas	4/124 (3)

*LFTs* Liver function tests, *VTE* Venous thromboembolism, *DKA* Diabetic ketoacidosis, *IFN* Interferon, *CT* Computed tomography

## Discussion

This is the first study to describe the content and quantity of internal medicine attending comments in morning report. Out of 2,344 observed virtual and in-person attending comments, we found that most comments were teaching-oriented, unprompted by facilitators, and brief (< 1 min). The most frequent teaching topics were differential diagnosis, management, and testing, which together accounted for almost half of all teaching comments. High-value care, patient safety, ethics, and social determinants of health were rarely discussed. Non-teaching comments were also common, and jokes were approximately as prevalent as comments about evidence appraisal. The frequency and duration of comments varied widely between sites. Variables that were associated with increased attending comments included report duration, in-person reports as opposed to virtual conferences, and the number of general internists. Scripted reports were associated with fewer comments.

Our results have relevance for medical educators in several ways. First, our observations of attending participation in morning report align with resident expectations: in a 2021 survey of internal medicine residents, Albert et al. found that residents preferred practical clinical teaching at morning report and that the top three teaching domains desired by residents were differential diagnosis, diagnostic work-up, and management [13]. These were found to be the three most prevalent categories of attending teaching comments in our study. Survey respondents also rated several non-clinical domains highly, including evidence-based medicine (EBM) and pathophysiology. These were less commonly mentioned by attendings, accounting for 5–6% of total comments. This may highlight an opportunity for improvement, suggesting that attendings should continue to discuss clinical topics and consider increasing content on EBM and pathophysiology. Other content areas that were rarely mentioned by attending physicians, including high value care, patient safety, and recognition of social determinants of health, also represent possible opportunities for improvement for residency programs who wish to increase focus on these subjects.

The second important finding from our study is the identification of variables that may impact attending participation. In-person conferences and unscripted reports were each associated with a considerably higher number of attending comments. Residency program leaders that are interested in increasing attending participation may consider utilizing these formats more frequently. The presence of general internists but not hospitalists or subspecialists also had a substantial association with increased number of attending comments. Conversely, virtual reports and scripted cases were associated with a reduced frequency of attending comments, and thus residencies that are interested in reducing attending participation may consider implementing these formats or increasing their use.

Our results do not clarify whether there is an ideal number of attending comments nor how much of a role attendings should have in morning report. This is an important consideration, as we suspect that the wide variation in the number of comments at each site may have a substantial effect on the learning environment. Moreover, the value of attending commentary in morning report likely varies institutionally according to program director, chief resident, or resident preferences. Conferences with few attending contributions, for instance, may reflect an educational experience driven by near-peers, but may also reflect an environment in which a few well-timed attending comments significantly affect the topic of conversation. Conversely, a conference dominated by attending commentary may comprise either a healthy pedagogy or an environment in which few trainees feel comfortable speaking. Rather than aspiring to a uniform ideal of attending involvement at morning report, stakeholders should identify local educational priorities and consider using our results to modify their conference.

The third important finding from our study is that attendings commented on a wide range of syndromes and diseases. No syndrome within differential diagnosis or management accounted for more than 6% of comments, while no specific disease accounted for more than 5% of differential comments. While an earlier report found that 42% of morning reports focused on rare diseases [12], attendings in our study commented on both common (e.g. hepatic encephalopathy) and rare (e.g. aortic dissection) pathology. Of note, discussion of coronavirus-associated illness was uncommon across all sites, suggesting that observed content of teaching points was similar to pre-pandemic settings. Importantly, the content of attending teaching comments is likely affected by numerous factors, including local prevalence of disease, attending level of expertise, facilitator choice of topic in scripted reports, and presenter choice of case in unscripted reports [10]. Reassuringly, our results demonstrated that attendings taught broadly across a range of topics and did not overwhelmingly teach towards one specific syndrome or diagnosis.

Our study has several limitations. First, the site investigators were all hospitalists which may have influenced our results about the types of attendings present. Second, all morning reports involved Veterans Health Administration (VHA) hospitals which may have influenced the types of cases discussed and thereby influenced attending comments. Third, we did not formally assess inter-rater reliability and many of the variables we assessed did require some reviewer judgement, such as whether comments were teaching or not. To minimize variation between observers we iteratively and collaboratively arrived at definitions and resolved discrepancies as a group through five training reports, as described in the methods. Fourth, our study was conducted during the COVID-19 pandemic which may have affected attending behavior, presence, and commentary in both in-person and virtual conferences. In particular, the virtual classroom may represent a radically different learning environment for both trainees and attending physicians and warrants additional study. Finally, we observed 250 reports at 13 sites which is a relatively small sample of morning reports at more than 500 internal medicine residency programs in the United States [18].

## Conclusions

Our study is the only multicenter description of comments made by attending physicians in morning report. We found that attending physicians’ comments focused on differential diagnosis, management, and testing which aligns with previously described resident learning goals. There were substantial differences in terms of the number of comments and their duration which likely affects the local learning environment. Morning report stakeholders that are interested in increasing attending involvement in morning report should consider encouraging in-person and unscripted reports, while educators that are interested in decreased attending involvement should consider virtual formats and scripted reports.

## Data Availability

Data analysis and data collection tools used in the current study are available from the corresponding author on reasonable request.

## Abbreviations

IRB: Institutional Review Board
VHA: Veterans Health Administration
EBM: Evidence-based medicine
